# Optimization of the Meat Flavoring Production Process for Plant-Based Products Using the Taguchi Method

**DOI:** 10.3390/foods14010116

**Published:** 2025-01-03

**Authors:** Varuzhan Sarkisyan, Anastasiya Bilyalova, Valentina Vorobyeva, Irina Vorobyeva, Alexey Malinkin, Vladimir Zotov, Alla Kochetkova

**Affiliations:** 1 Laboratory of Food Biotechnology and Foods for Special Dietary Uses, Federal State Budgetary Scientific Institution Federal Research Center of Nutrition, Biotechnology and Food Safety, 109240 Moscow, Russia; 2 Laboratory of Food Chemistry, Federal State Budgetary Scientific Institution Federal Research Center of Nutrition, Biotechnology and Food Safety, 109240 Moscow, Russia

**Keywords:** meat flavoring, Taguchi method, sensory characteristics, volatile organic compounds

## Abstract

The development of plant-based meat substitutes is imperative for reducing animal fat intake and promoting dietary diversification. However, the flavor profiles of these products frequently fall short of consumer expectations. This study sought to optimize the production process of meat flavorings for plant-based products using the Taguchi method. The study investigated the effects of sugar type, concentration, and reaction temperature on the Maillard reaction products, sensory characteristics, and volatile organic compounds. The thermal process flavors were obtained from the flavor precursor by heating in a laboratory microwave station at 30 bar for 15 min. The variable factors were the type of sugar (fructose, glucose, xylose), its concentration (25, 50, and 100 mM), and the temperature of the reaction (140, 150, and 160 °C). The study’s findings indicated that temperature emerged as the predominant factor influencing the formation of Maillard reaction products and the sensory characteristics of the flavorings. Specifically, 25 mM xylose-based flavorings prepared at 140 °C demonstrated the most notable meat flavor and the highest level of acceptability. Moreover, the analysis of volatile organic compounds revealed the presence of a diverse array of substances, including aldehydes, ketones, and alcohols, that are characteristic of meat flavor. A heat map of the volatile content was constructed to facilitate a comparison of the samples. The study demonstrates the effectiveness of the Taguchi method in optimizing the production process of meat flavorings for plant-based products and provides valuable insights for the development of more balanced odor profiles.

## 1. Introduction

The development of plant-based meat substitutes may contribute to reducing the traditionally high intake of animal fats and facilitate dietary diversification among individuals who do not consume animal products. The primary challenges associated with the development of these products pertain to their flavor profiles, which are often perceived as unpleasant and may be influenced by intrinsic and extrinsic factors. This can lead to a lower consumer acceptance of these products. In its natural state, meat is characterized by a neutral flavor profile. However, meat analogs are products that emulate the final processed meat products, such as cutlets, schnitzels, and sausages. It is, therefore, essential not only to disguise any extraneous flavors and aromas but also to imbue the product with an organoleptic profile that is familiar to the consumer.

The use of flavoring agents plays an instrumental role in addressing a multitude of technological challenges inherent to the production of food products. For instance, the use of a flavoring agent enables the attainment of a desired sensory profile, which would otherwise be unattainable through alternative means. Additionally, it facilitates the restoration of lost or diminished aromas and flavors resulting from processing or storage. The creation of a diverse range of food products based on a single ingredient is a further advantage. Furthermore, the standardization of aroma and taste characteristics allows for greater control over the quality and consistency of the finished product, regardless of the quality of the raw materials used.

Food flavorings can be obtained by a variety of methods, including physical, enzymatic, and microbiological processes, from raw materials of plant, microbial, or animal origin. These materials may also be derived from traditional methods of food preparation [1]. The classification of flavoring agents is dependent on the method of obtaining and the purpose of flavoring application. They can be classified as flavoring precursors or natural or thermal process flavors.

A thermal process flavor is defined as a mixture of substances that have been produced as a consequence of the interaction between amino compounds and reducing sugars in the Maillard reaction within the specified technological parameters of the synthesis. The Maillard reaction is a non-enzymatic darkening reaction that plays a pivotal role in the formation of volatile flavor compounds and the appearance of cooked meat. The reaction products resulting from the thermal degradation of sugars and amino acids are contingent upon several factors, including the mixture heating temperature, the pH of the medium, the duration of the mixture processing, and the concentration of reacting components. The mechanism of the Maillard reaction consists of three stages. The first stage involves a reaction between reducing sugars and amino acids with further rearrangement to form Amadori products in the presence of aldose sugar or Haynes products in the presence of ketose sugar. In the second stage, rearrangement of Amadori or Haynes products occurs. In this stage, the sugars are broken down and release amino compounds. In the last stage, the amino compounds undergo dehydration, decomposition, cyclization, and polymerization. A number of aromatic compounds such as ketones, aldehydes, alcohols, furans, and their derivatives are formed [2]. These aromatic compounds constitute the meat flavor produced by the Maillard reaction.

Protein hydrolysates of plant or microbial origin are commonly used as a source of amino acids [3,4]. Various mono- and disaccharides are used as sources of reducing sugars, for many of which the peculiarities of participation in the formation of Maillard reaction products with the formation of specific fragrant substances have been studied [5]. It is characteristic that hexoses (glucose, fructose, and mannose) react faster than pentoses (ribose, xylose), which, in turn, are more reactive than disaccharides (lactose, maltose, sucrose) [6]. Both pentoses and hexoses can be represented by aldoses (glucose, xylose, ribose, mannose) and ketoses (fructose, ribulose). From the above list, three monosaccharides are the most commonly used: glucose (aldohexose), fructose (ketohexose), and xylose (aldopentose), which are promising precursors for meat flavorings [7,8].

In addition to low reactivity, disaccharides, when involved in the melaidin formation reaction, form more carcinogenic and mutagenic compounds compared to monosaccharides [9]. In this connection, their use in the development of meat flavorings is not reasonable. The formation of toxic products during thermal processing is an inevitable process, which, however, can be controlled by changing the technology [10]. The development of meat flavorings is a multifactor optimization problem and, in a traditional research design, involves the use of a large number of samples to set up a full factorial experiment [11]. It is possible to reduce the number of samples required for the study by using a fractional factor plan, in particular, the Taguchi method [12]. Such experimental plans have proven themselves in the optimization of processes in the food industry [13]. Taguchi’s method was applied to optimize a method for estimating the volatile organic compounds of a meat flavoring agent [14]. However, this method has not been applied to directly optimize the flavoring process.

The aim of the present work was to optimize the production process of meat flavorings for plant-based products using the Taguchi method.

## 2. Materials and Methods

### 2.1. Materials

To obtain a flavoring with a meat organoleptic profile, the present study used isolated soy protein with a protein content of at least 90% and yeast of the genus *Sacchoromyces cerevisiae* purchased from a local store in Moscow, Russia; chicken nuggets (Miratorg, Moscow, Russia); chemically pure crystalline fructose, crystalline hydrated glucose, crystalline xylose, and citric acid; distilled water.

### 2.2. Preparation of Yeast Autolysate

The principle of autolysate production is based on thermo-acid autolysis of a yeast cell biomass of the genus *S. cerevisiae* (“Rekord Red”, pressed yeast, Lesaffre, Voronezh, Russia), resulting in the cleavage of cell contents into nucleotides, amines, and monosaccharides. The composition of the medium for autolysis included pressed baker’s yeast and distilled water, and the ratio of components was 3:2 *w*/*w*. The pH of the medium was set in the range of values 3–3.2 using 50% (*w*/*w*) aqueous citric acid solution. The dry matter content of the medium was 10–15%. Thermo-acid autolysis was carried out in a GLS 80 heated reactor (DURAN, London, UK) at autolysis temperature (70 ± 2) °C with a stirring speed of 200 rpm for 48 h. After the set time, the autolysate was cooled to (20 ± 2) °C, and the biomass was separated on an Avanti JHC centrifuge (Beckman-Coulter, Brea, CA, USA) at 3000 rpm for 20 min. The obtained supernatant was yeast autolysate and was further used as a component of the nutrient medium.

The technological process of flavoring agent production with a meat organoleptic profile consists of two stages. In the first stage, the flavoring precursor from autolysate was obtained; in the second stage, the thermal process flavor from the precursor was obtained.

### 2.3. Preparation of Flavoring Precursor

The flavoring precursor was obtained by culturing *S. cerevisiae* (Dr. Oetker, Belgorod, Russia) yeast in a GLS 80 reactor (Duran, UK) in liquid nutrient medium at (30 ± 0.5) °C for 24 h. The medium was composed of the following components: yeast autolysate (420 mL), soy protein isolate (60 g), crystalline glucose (15 g), L-cysteine (3 g), and distilled water (up to 1000 mL). The pH of the medium was adjusted to 5.0–5.5 using a 20% (*w*/*w*) aqueous sodium hydroxide solution. After completion of the cultivation process, the obtained product was centrifuged at 3000 rpm for 20 min and the supernatant with a dry matter content of at least 10% was separated.

### 2.4. Preparation of Thermal Process Flavor

The Taguchi method was used in planning the experiment to reduce the number of experiments required to analyze the effect of various factors.

The thermal process flavor was obtained from the precursor by heating in an Ultrawave microwave laboratory station (Milestone Srl, Milano, Italy) at 30 bar for 15 min. The variable factors were the type of sugar (fructose, glucose, xylose), their concentration (25 mM, 50 mM, and 100 mM), and the reaction temperature (140 °C, 150 °C, and 160 °C). In this regard, the L9 orthogonal array (3 factors with three levels) was applied. The composition and mode of technological treatment of samples (S) used in the experiment are summarized in Table 1.

### 2.5. Sensory Analysis

Sensory analysis of technical thermal flavorings was conducted by trained experts who demonstrated satisfactory odor detection and recognition thresholds in accordance with the ISO 5496:2006 standard [15]. The study was conducted in the test room intended for sensory analysis according to ISO 8589:2007 [16]. Ten experts (four men and six women, aged 28 to 69 years) were selected for the sensory evaluation. Panelists had at least 5 years of experience in sensory analysis. The panel sessions were held at mid-morning, about 3 h after breakfast. Equal volumes of flavor samples were placed in glass beakers and presented to the tasters for evaluation.

Three descriptors were selected as a response quality characteristic of the Taguchi model: cooked meat odor (larger-the-better), off-odor (smaller-the-better), and acceptance (larger-the-better). The cooked meat odor was evaluated using a hedonic scale by comparing it to the odor of a cooked chicken breast sample (fried 1 h prior to study on a preheated pan at 180 °C for 10 min with sunflower oil) as a reference sample [17]. The off-odor is an odor atypical of cooked meat that is associated with spoilage or a change in the chemical composition of the product (according to ISO 5492:2008 [18]). For evaluating acceptance, panelists were asked to give a semihedonic quality score based on their expertise [19]. The descriptors were evaluated by inhaling the vapor phase of the samples.

In addition to the study conducted, a meat product analog containing the most preferred flavoring (namely S8) was developed. To obtain 100 g of the product, 14 g of textured soy protein (with a particle size ranging from 0.5 to 1 cm) was utilized, which was ground on a cutter in 47.6 g of a water–ice mixture (at a temperature of 8 °C) with 2.8 g of calcium gluconate and 1 g of sodium chloride. Separately, 15.4 g of sunflower oil was combined with 9.2 g of citrus dietary fiber, yielding a suspension of fibers in oil. This mixture was introduced into the cutter, where it was mixed until homogeneous. After that, 10 g of S8 was added to the mixture. The resulting mixture was shaped into molds typical of nuggets, with a weight of 20–25 g each. The molded products were then dipped sequentially in egg wash and breadcrumbs. The products were then subjected to a frying process in a pan with sunflower oil at a temperature of 180 °C for a duration of 10 min. The odor of the meat analogs was then compared with that of commercial chicken. The members of the sensory panel conducted a discussion regarding the odor descriptors of the samples, which included such terms as “cooked meat”, “caramel”, “roasted”, “popcorn”, “mushroom”, “bread”, and “floral”. The panel members were trained for this analysis according to a procedure described in [20].

A ten-point (0–2, weaker; 3–4, weak; 5–6, middle; 7–8, strong; 9–10, stronger) universal intensity scale was applied to all sensory studies [21].

### 2.6. Determination of Maillard Reaction Products Content

Accumulation of Maillard reaction products was measured using a spectrofluorimetric method similar to the method of Yamaguchi et al. with modifications [22]. The samples were diluted 10 times with distilled water and measured at 333/425 nm (ex./em.), characteristic for furosine fluorescence in a quartz cuvette with a path length of 1 cm on an Agilent Cary Eclipse spectrofluorimeter (Agilent Technologies, Santa Clara, CA, USA).

### 2.7. Analysis of the Profile of Volatile Organic Components

The reaction products were analyzed by GC with a vapor phase injection system and mass spectrometry. To investigate the composition of fragrance substances of the obtained fragrances, a fiber for solid-phase microextraction coated with divinylbenzene/carboxene/polydimethylsiloxane 50/30 μm (Supelco, Bellefonte, PA, USA) was used. Prior to analysis, the fiber was conditioned according to the manufacturer’s recommendations. An Agilent Technologies 7890A gas chromatograph (Agilent, Santa Clara, CA, USA) with flame ionization detector, Agilent Technologies 7000B mass detector (USA), and Supelcowax 10 60 m × 0.53 mm × 1 μm chromatography column (Supelco, USA) were used. The following temperature program was used for the Supelcowax 10 column: 35 °C for 5 min, heating to 220 °C at a rate of 4 °C/min, isotherm for 50 min. The carrier gas was helium, mode without flow division, injector temperature 225 °C. Mass detector operation parameters were scanning range 35–400 *m*/*z*, ionization source temperature 230 °C, quadrupole temperature 150 °C, and ionization by electron impact with energy 70 eV. A sample suspension weighing 2.000 ± 0.100 g was introduced into a vial for vapor phase analysis; 30.0 ± 0.5 mg of internal standard solution (aqueous solution of nonanone-5 with a concentration of 8.77 μg/g) was added. The vial was sealed with a septum cap, and the vial was thermostated at 50 °C with stirring for 20 min. The preconditioned fiber for analysis by solid-phase microextraction was then placed in the space above the sample, conditioned for 10 min, and then placed in the chromatograph injector for 5 min, and chromatographic analysis was performed. Fiber conditioning, extraction, and sample injection were performed using an automated sample preparation station, Gerstel MPS (Gerstel, Mülheim an der Ruhr, Germany).

Peak identification was performed by comparing the obtained mass spectra with library mass spectra using the programs “Agilent MassHunter Qualitative Analysis Navigator B.08.00”, “NIST MS Search 2.0”, and “MS-DIAL ver. 4.9.221218” and by comparing the calculated Kovacs retention indices with the index values presented in the PubChem database (index values for polar columns). Compounds with a mass spectrum match factor (Match value) of at least 650 were considered to be identified. All samples were analyzed in 3 repetitions.

### 2.8. Toxicity Assessment

Virtual screening of toxicity classes of volatile organic compounds included in the profile of the developed flavorings was carried out using the ProTox server (version 3.0) according to standard protocols [23].

### 2.9. Statistical Analysis

Statistical analysis of the obtained results was performed in the RStudio (version: 2023.06.1 Build 524, R version 4.2.2) environment [24] using ggplot2 (version: 3.4.1) [25] and tidyverse (version: 2.0.0) [26] packages with the selection of optimal technological parameters using the factor effects diagram for sensitivity. When comparing between-group effects for fluorescence index, one-factor ANOVA and Tukey’s post-hoc test for pairwise comparison were applied. When comparing the effect in the results of sensory analysis (categorical data), the Kruskal–Wallis test was used with Dunn’s test for post-hoc.

## 3. Results and Discussion

**Figure 1:**
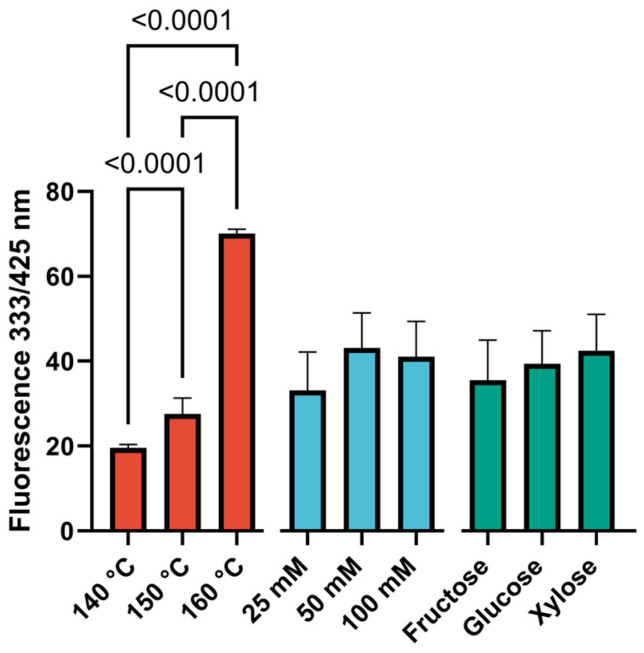
Diagrams of the effects of factors on Maillard reaction product content as a function of temperature, concentration, and monosaccharide type. Data are presented as the median ± 95% confidence interval. N = 24 for each bar.

The accumulation of Maillard reaction products is analyzed in Figure 1. As can be seen from the figure, the temperature factor has the greatest contribution to the intensity of the formation of reaction products.

The obtained data indicate that temperature is the most significant factor influencing the rate of formation of Maillard reaction products. This finding is consistent with the results reported by Sun et al. [27] and Chen et al. [28]. A statistically significant difference between the groups was observed for this index (*p* < 0.0001). The intensity of formation of Maillard reaction products varies depending on the type of monosaccharide, with fructose exhibiting the lowest intensity, followed by glucose and then xylose. This dependence is analogous to the findings reported in Laroque et al. [29]. Despite the low dispersion of fluorescence analysis results in the studied samples (due to the greater influence of temperature), the contribution of reducing sugars to the development of the Maillard reaction is significant. The type of sugar determines not only the rate of formation of reaction products but also the diversity of these products, providing differences in the profiles of volatiles [30].

Furthermore, the data demonstrate that the quantity of reaction products increases in conjunction with the rising concentration of reducing sugars in the mixture (Figure 1). This suggests that, within the confines of the experimental parameters, the formation of reaction products can be described as a non-zero-order reaction. This is consistent with observations of analogous processes occurring in disparate food systems [31]. Furthermore, the accumulation of Maillard reaction products was determined by the fluorescence wavelength characteristic of furosine, a product of the reaction of l-lysine with sugars. The accumulation of furosine is also consistent with a zero-order kinetic model, indicating that it is independent of the initial substance concentration. Consequently, it is more appropriate to discuss not the change in the reaction rate with increasing concentration of reducing sugars but rather the completeness of the reaction. This is due to the fact that the lysine content is constant across all samples and is determined by its content in the yeast substrate [32]. The utilization of monosaccharide concentrations exceeding 50 mM is not reasonable, as it does not result in a discernible increase in fluorescence values.

**Figure 2:**
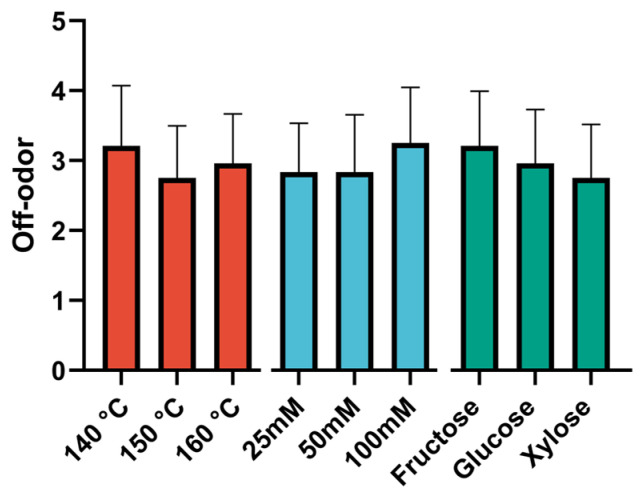
Diagrams of the effects of sugar type on off-odor. Data are presented as the median ± 95% confidence interval. N = 24 for each bar.

**Figure 3:**
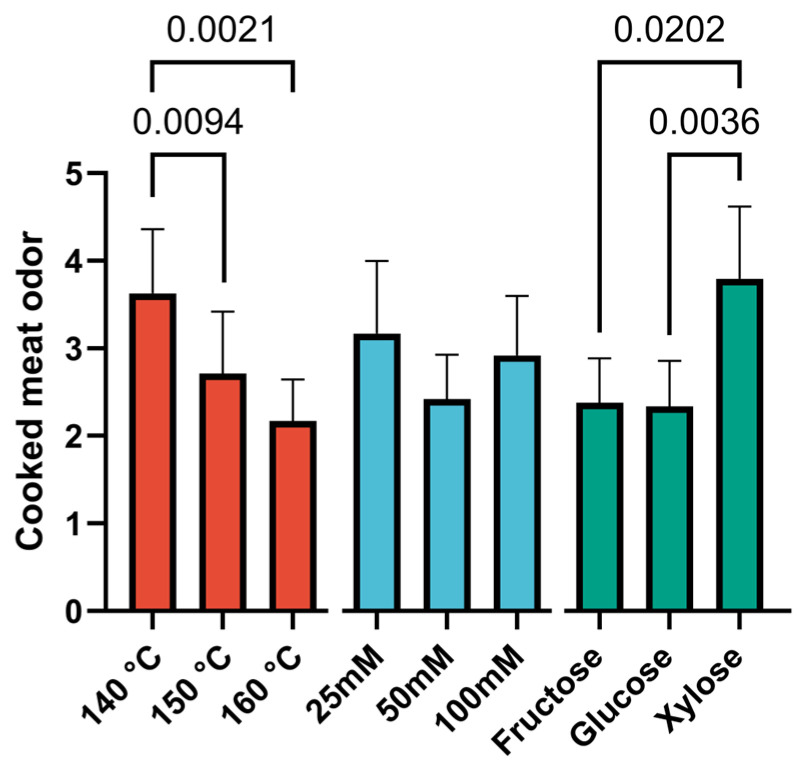
Diagrams of the effects of processing temperature on cooked meat odor. Data are presented as the median ± 95% confidence interval. N = 24 for each bar.

**Figure 4:**
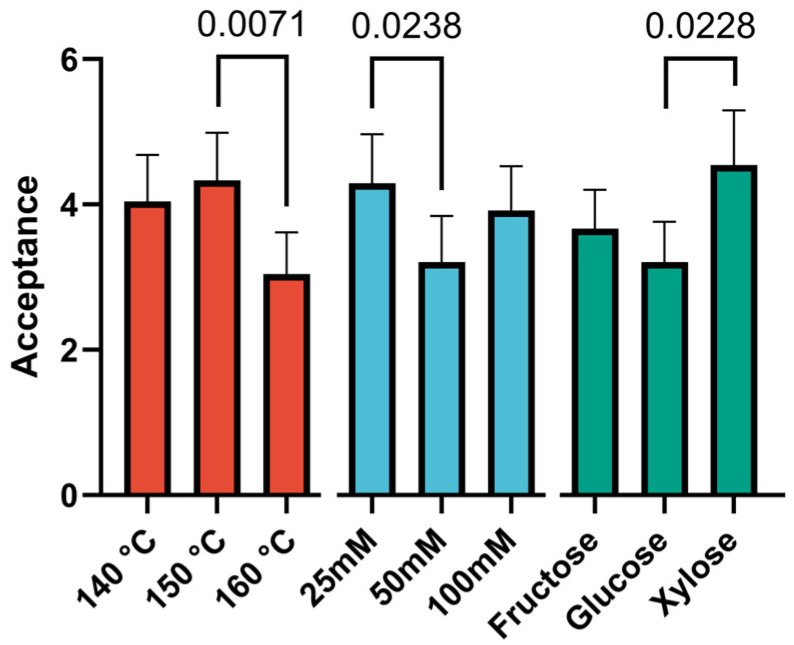
Diagrams of the effects of sugar concentration on acceptance. Data are presented as the median ± 95% confidence interval. N = 24 for each bar.

The results of the sensory analysis, presented in the form of diagrams illustrating the effects of the studied factors, are shown in Figure 2, Figure 3 and Figure 4.

This parameter demonstrated a lesser degree of dependence on the selected factors and exhibited stability with respect to the selected factors. No statistically significant differences were observed between the groups for any of the factors (*p* > 0.05). This may be attributed to the influence of an additional factor common to all groups, in addition to the selected factors, on the expression of foreign odor. In this case, the type of monosaccharide used had the greatest effect. Samples with fructose exhibited the most pronounced extraneous odor, while those with xylose demonstrated the least. The panelists were queried about which descriptors would most aptly characterize the extraneous odor of the samples. The most frequently mentioned descriptors were “popcorn”, “mushroom”, and “floral”.

The odor of the cooked meat was highly dependent on two factors: temperature and type of saccharide (Figure 3). The best evaluation was obtained at a lower temperature (140 °C). Significant differences were found with both samples cooked at 150 °C (*p* = 0.0094) and 160 °C (*p* = 0.0021). The most pronounced flavor of cooked meat is also characteristic of samples containing xylose, significantly different from glucose (*p* = 0.0036) and fructose (*p* = 0.0202) flavoring samples.

The combined organoleptic characteristics also influenced the overall acceptability of the flavor (Figure 4).

The acceptability values exhibited considerable variability across all factors. It is noteworthy that, although the most pronounced meat flavor is characteristic at 140 °C, samples obtained at 150 °C are rated the highest in terms of preference. This may be attributed to the diminished expression of extraneous odors at this temperature. The results for the factors of sugar concentration and type of sugars are analogous to those for the intensity of meat flavor, with samples containing 25 mM sugars being the most preferred and xylose being the most preferred among these samples.

To elucidate the discrepancies in the expression of meat and foreign odor in the studied samples, the profile of volatile organic substances was analyzed. A comprehensive list of the identified compounds is provided in Table 2.

The majority of the identified substances are indicative of a meat flavor profile. In particular, the most pronounced meat odors are as follows. Additionally, 2-methylthiophene, thiazole, 2-pentylthiophene, and 2-thiophenecarboxaldehyde were identified in the samples. However, the aroma of cooked or roasted meat is not solely constituted by the principal meat aroma note but also by a complex of associated odors. Some of the substances responsible for the formation of these aromas were identified in the samples.

The aldehydes characteristic of the meat aroma were identified as nonanal (fatty, floral, waxy odor), benzaldehyde (almond, burnt sugar odor), and heptanal (nutty, fruity, green odor) [33,34]. The most characteristic representatives of ketones were 2-butanone (chemical, burnt, gas, chocolate odor) and 2-heptanone (citrus, grapefruit, floral, fruity, spicy, cinnamon odor) [35,36]. Additionally, the samples exhibited the presence of alcohols that are characteristic of meat fat oxidation processes during heat treatment. Additionally, 1-octen-3-ol (characterized by a mushroom odor), 1-hexanol (perceived as having woody, cut grass, chemical, wine, fatty, fruity, and weak metallic qualities), and 1-heptanol (with a floral odor) were identified as well as ethanol (which has been associated with the aroma of alcohol and white wine) [37,38,39].

In addition to the substances that are characteristic of the meat flavor, volatile compounds have been identified as a potential source of off-flavor formation in flavorings. These include acetaldehyde, which has a pungent, choking odor when concentrated and a fruity odor when diluted; nitrobenzene, which has a bitter almond odor; benzene acetaldehyde, which has a green and floral odor; and hexyl acetate, which has a fruit and herbal odor.

**Figure 5:**
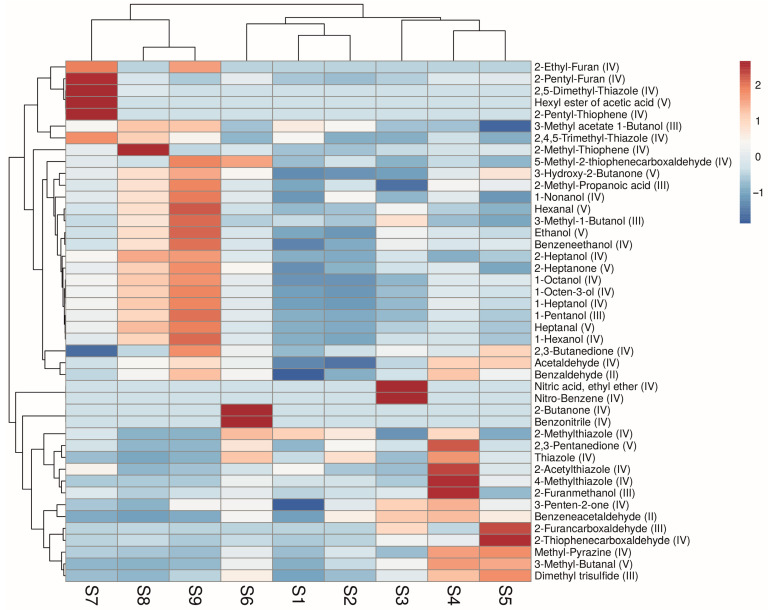
Heat map of the volatile content of the samples (the predicted toxicity class of the substance is indicated in parentheses; data are centered and scaled by row; Pearson distance was used to construct dendrograms).

To facilitate a comparison of the studied samples, a heat map of the volatile content was constructed (Figure 5). As illustrated in the figure, the most toxic substances identified (toxicity class II) are benzaldehyde and benzenacetaldehyde. Seven compounds belong to class III (3-methylacetate, 2-methylpropionic acid, 3-methyl-1-butanol, 1-pentanol, 2-furanmethanol, 2-furancarboxaldehyde, and dimethyltrisulfide), while the remaining substances exhibit lower toxicity.

As with the outcomes of the Myre reaction products and sensory analysis, the processing temperature had the most significant impact. The highest concentration of volatile compounds with a distinctive meat-like aroma was observed in samples subjected to thermal treatment at 140 °C (S7–S9). The samples obtained at 150 °C and 160 °C exhibited a diminished concentration of meat aroma substances and an elevated presence of burnt and off-odor substances.

Nevertheless, notable discrepancies exist even between samples obtained at 140 °C. The most distinctive profile was observed in sample S7 (140 °C, fructose, 50 mM). The highest concentrations of 2-ethylfuran, 2-pentylfuran, 2,5-dimethylthiazole, hexyl acetate, and 2-pentylthiophene were observed, while the lowest levels of 2,3-butandienone were present.

Samples S8 (140 °C, xylose, 25 mM) and S9 (140 °C, glucose, 100 mM) exhibited notable similarities, particularly in the profiles of alcohols, aliphatic aldehydes, and ketones, which were higher in sample S9 than in S8. The distinctive feature of sample S8 is its elevated concentration of 2-methyl-thiopene, which imparted a pronounced meat-like aroma. These findings align with prior observations regarding flavorings derived from yeast and fructose hydrolysates in the presence of cysteine [40].

**Figure 6:**
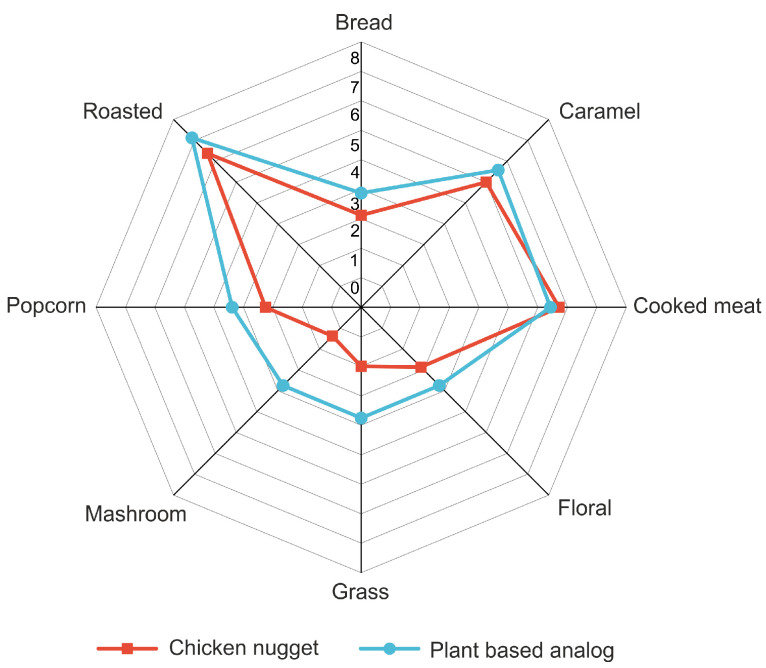
Odor profiles for chicken nuggets and plant-based analogs.

Using sample S8, a plant-based meat analog product was developed, and its aroma profile was compared to that of chicken nuggets. Figure 6 shows the radar plots for the sensory analysis. The findings of the sensory analysis suggest that the primary profile descriptors delineating the meat flavor of the products (i.e., roasted and cooked meat) exhibit no significant disparities.

The most significant differences were found for the descriptors characterizing extraneous odor, including popcorn (*p* > 0.05), mushroom (*p* < 0.01), grass (*p* < 0.05), and caramel (*p* > 0.05). Of the aforementioned descriptors, mushroom, caramel, and popcorn were identified for the flavor samples directly. The emergence of novel descriptors, such as “grassy” and “floral”, is likely associated with the flavor of plant product components, particularly soybean texturate. The manifestation of these descriptors is characteristic of plant analogs of meat products [20]. It is plausible that this is attributable to the flavor of plant product components, particularly soy texturates. The sensory profile of meat products is characterized by a distinctive mushroom odor; however, the incorporation of a flavoring agent resulted in a substantial enhancement of this odor. The presence of mushroom odor in the product may also be attributable to the high content of 1-octen-3-ol in the composition of flavoring S8 [41].

In addition to the aforementioned descriptors, a pronounced odor of bread is evident, associated with the presence of breadcrumbs in both samples. No significant differences in the value of this descriptor were observed (*p* > 0.05). The obtained results generally indicate the feasibility of producing new types of plant analogs of meat products with a pronounced meat flavor by using the developed flavoring agents.

## 4. Conclusions

The results of the conducted studies indicate that the Taguchi method can be effectively applied to optimize the planning of experiments for the purpose of obtaining meat flavorings for non-meat products. The results demonstrate that the processing temperature is the most significant factor influencing the organoleptic characteristics of the flavoring. The most acceptable meat flavor can be achieved by utilizing 25 mM xylose as a source of reducing sugars in the production of technical thermal flavoring. An essential objective is to conduct further research to identify the factors causing the manifestation of extraneous odors and to develop methods for their correction, with the aim of obtaining more balanced flavor profiles of flavoring agents. The plant analog of chicken nuggets developed using this flavoring agent was characterized by a distinct meat flavor. The findings of the present study are constrained by the modest size of the panel of expert tasters and can be expanded upon by incorporating the results of the study of sensory properties of herbal products with meat flavorings within the context of consumer studies.

## Figures and Tables

**Table 1:** Characteristics of the samples.

Sugar	Concentration, mM	Temperature, °C	Sample Number
Fructose	25	150	1
Fructose	50	140	7
Fructose	100	160	4
Xylose	25	140	8
Xylose	50	160	5
Xylose	100	150	2
Glucose	25	160	6
Glucose	50	150	3
Glucose	100	140	9

**Table 2:** Information on the content of volatile organic substances in technical thermal flavorings.

Substance	S1	S2	S3	S4	S5	S6	S7	S8	S9
Acetaldehyde	0.098 ± 0.002 ^c^	0.062 ± 0.004 ^c^	0.244 ± 0.022 ^a^	0.495 ± 0.035 ^b^	0.505 ± 0.000 ^b^	0.339 ± 0.004 ^b^	0.255 ± 0.004 ^a^	0.374 ± 0.004 ^b^	0.458 ± 0.011 ^b^
2-Butanone	0.000 ± 0.000 ^a^	0.000 ± 0.000 ^a^	0.000 ± 0.000 ^a^	0.000 ± 0.000 ^a^	0.000 ± 0.000 ^a^	0.01 ± 0.001 ^a^	0.000 ± 0.000 ^a^	0.000 ± 0.000 ^a^	0.000 ± 0.000 ^a^
3-Methyl-Butanal	0.069 ± 0.009 ^a^	0.514 ± 0.017 ^b^	1.081 ± 0.013 ^c^	2.197 ± 0.249 ^d^	2.079 ± 0.052 ^d^	1.005 ± 0.027 ^c^	0.159 ± 0.019 ^b^	0.129 ± 0.015 ^b^	0.176 ± 0.019 ^b^
Ethanol	5.662 ± 0.742 ^d^	4.935 ± 0.475 ^d^	10.837 ± 0.905 ^c^	9.146 ± 0.739 ^c^	8.236 ± 1.163 ^c^	9.146 ± 0.495 ^c^	8.5 ± 0.558 ^c^	13.571 ± 1.337 ^b^	18.926 ± 1.773 ^a^
2-Ethyl-Furan	0.000 ± 0.000 ^a^	0.000 ± 0.000 ^a^	0.000 ± 0.000 ^a^	0.000 ± 0.000 ^a^	0.000 ± 0.000 ^a^	0.000 ± 0.000 ^a^	0.008 ± 0.001 ^a^	0.000 ± 0.000 ^a^	0.007 ± 0.001 ^a^
Nitric acid, ethyl ether	0.000 ± 0.000 ^a^	0.000 ± 0.000 ^a^	0.044 ± 0.001 ^a^	0.000 ± 0.000 ^a^	0.000 ± 0.000 ^a^	0.000 ± 0.000 ^a^	0.000 ± 0.000 ^a^	0.000 ± 0.000 ^a^	0.000 ± 0.000 ^a^
2,3-Butanedione	0.047 ± 0.007 ^a^	0.067 ± 0.01 ^b^	0.097 ± 0.01 ^c^	0.078 ± 0.009 ^b^	0.138 ± 0.019	0.095 ± 0.002 ^c^	0.000 ± 0.000 ^a^	0.063 ± 0.000 ^b^	0.177 ± 0.021 ^d^
2,3-Pentanedione	0.000 ± 0.000 ^a^	0.103 ± 0.001 ^c^	0.045 ± 0.004 ^b^	0.26 ± 0.026 ^d^	0.041 ± 0.004 ^b^	0.131 ± 0.013 ^c^	0.047 ± 0.003 ^b^	0.000 ± 0.000 ^a^	0.000 ± 0.000 ^a^
Hexanal	0.211 ± 0.007 ^a^	0.232 ± 0.025 ^a^	0.408 ± 0.035 ^b^	0.256 ± 0.011 ^a^	0.193 ± 0.008 ^a^	0.286 ± 0.017 ^a^	0.318 ± 0.042 ^b^	0.506 ± 0.063 ^b^	0.768 ± 0.104 ^c^
2-Methyl-Thiophene	0.006 ± 0.001 ^a^	0.007 ± 0.001 ^a^	0.053 ± 0.005 ^b^	0.072 ± 0.005 ^c^	0.095 ± 0.002 ^d^	0.085 ± 0.008 ^c^	0.123 ± 0.007 ^e^	0.547 ± 0.047 ^f^	0.043 ± 0.000 ^b^
3-Methyl acetate 1-Butanol	0.017 ± 0.000 ^b^	0.016 ± 0.002 ^b^	0.011 ± 0.000 ^b^	0.011 ± 0.001 ^b^	0.006 ± 0.001 ^a^	0.011 ± 0.000 ^b^	0.016 ± 0.002 ^b^	0.02 ± 0.000 ^c^	0.02 ± 0.002 ^c^
3-Penten-2-one	0.002 ± 0.000 ^a^	0.031 ± 0.003 ^b^	0.049 ± 0.001 ^c^	0.054 ± 0.007 ^c^	0.035 ± 0.003 ^b^	0.037 ± 0.005 ^b^	0.022 ± 0.001 ^b^	0.018 ± 0.000 ^b^	0.037 ± 0.004 ^b^
2-Heptanone	0.026 ± 0.002 ^a^	0.038 ± 0.004 ^a^	0.053 ± 0.005 ^b^	0.06 ± 0.004 ^b^	0.034 ± 0.001 ^a^	0.075 ± 0.007 ^b^	0.065 ± 0.006 ^b^	0.096 ± 0.006	0.115 ± 0.002 ^c^
Heptanal	0.006 ± 0.001 ^a^	0.005 ± 0.001 ^a^	0.01 ± 0.001 ^b^	0.012 ± 0.001 ^b^	0.009 ± 0.000 ^a^	0.014 ± 0.000 ^b^	0.018 ± 0.001 ^b^	0.031 ± 0.003 ^c^	0.038 ± 0.001 ^c^
3-Methyl-1-Butanol	0.792 ± 0.022 ^b^	0.687 ± 0.042 ^a^	1.346 ± 0.072 ^c^	0.662 ± 0.098 ^a^	0.521 ± 0.000 ^a^	0.736 ± 0.107 ^b^	0.796 ± 0.058 ^b^	1.316 ± 0.082 ^c^	1.931 ± 0.115 ^d^
2-Pentyl-Furan	0.058 ± 0.008 ^c^	0.042 ± 0.005 ^c^	0.087 ± 0.009 ^c^	0.215 ± 0.011 ^b^	0.227 ± 0.005 ^b^	0.248 ± 0.02 ^b^	0.934 ± 0.025 ^a^	0.19 ± 0.008 ^b^	0.079 ± 0.008 ^c^
1-Pentanol	0.011 ± 0.001 ^a^	0.009 ± 0.000 ^a^	0.016 ± 0.002 ^a^	0.037 ± 0.005 ^b^	0.023 ± 0.001 ^a^	0.043 ± 0.006 ^b^	0.05 ± 0.002 ^b^	0.079 ± 0.011 ^c^	0.116 ± 0.01 ^d^
2-Methylthiazole	0.02 ± 0.001 ^c^	0.016 ± 0.000 ^b^	0.000 ± 0.000 ^a^	0.019 ± 0.000 ^c^	0.002 ± 0.000 ^a^	0.022 ± 0.003 ^c^	0.009 ± 0.001 ^a^	0.003 ± 0.000 ^a^	0.003 ± 0.000 ^a^
Thiazole	0.095 ± 0.011 ^b^	0.195 ± 0.015 ^a^	0.071 ± 0.008 ^c^	0.263 ± 0.029 ^a^	0.118 ± 0.009	0.226 ± 0.03 ^a^	0.07 ± 0.003 ^c^	0.051 ± 0.001 ^d^	0.059 ± 0.006 ^d^
Hexyl ester of acetic acid	0.000 ± 0.000 ^a^	0.000 ± 0.000 ^a^	0.000 ± 0.000 ^a^	0.000 ± 0.000 ^a^	0.000 ± 0.000 ^a^	0.000 ± 0.000 ^a^	0.004 ± 0.000 ^a^	0.000 ± 0.000 ^a^	0.000 ± 0.000 ^a^
Methyl-Pyrazine	0.009 ± 0.000 ^a^	0.202 ± 0.012 ^b^	0.002 ± 0.000 ^a^	0.868 ± 0.067 ^c^	0.928 ± 0.053 ^c^	0.284 ± 0.002 ^b^	0.062 ± 0.003 ^a^	0.031 ± 0.002 ^a^	0.001 ± 0.000 ^a^
4-Methylthiazole	0.005 ± 0.000 ^a^	0.005 ± 0.000 ^a^	0.000 ± 0.000 ^a^	0.05 ± 0.004 ^b^	0.011 ± 0.000 ^c^	0.012 ± 0.001 ^c^	0.000 ± 0.000 ^a^	0.000 ± 0.000 ^a^	0.000 ± 0.000 ^a^
3-Hydroxy-2-Butanone	0.001 ± 0.000 ^c^	0.006 ± 0.000 ^c^	0.013 ± 0.001 ^c^	0.076 ± 0.007 ^b^	0.123 ± 0.014 ^b^	0.104 ± 0.006 ^b^	0.084 ± 0.001 ^b^	0.131 ± 0.015 ^b^	0.172 ± 0.01 ^a^
2-Heptanol	0.011 ± 0.000 ^a^	0.01 ± 0.000 ^a^	0.02 ± 0.002 ^b^	0.011 ± 0.001 ^a^	0.015 ± 0.002 ^a^	0.022 ± 0.000 ^b^	0.028 ± 0.001 ^b^	0.043 ± 0.004 ^c^	0.045 ± 0.000 ^c^
2,5-Dimethyl-Thiazole	0.000 ± 0.000 ^a^	0.000 ± 0.000 ^a^	0.000 ± 0.000 ^a^	0.000 ± 0.000 ^a^	0.000 ± 0.000 ^a^	0.000 ± 0.000 ^a^	0.031 ± 0.001 ^b^	0.003 ± 0.000 ^a^	0.000 ± 0.000 ^a^
1-Hexanol	0.381 ± 0.045	0.346 ± 0.001	0.686 ± 0.035	0.729 ± 0.052	0.539 ± 0.078	0.829 ± 0.047	0.896 ± 0.109	1.465 ± 0.188	2.033 ± 0.084
2,4,5-Trimethyl-Thiazole	0.014 ± 0.001 ^b^	0.000 ± 0.000 ^a^	0.000 ± 0.000 ^a^	0.006 ± 0.000 ^a^	0.000 ± 0.000 ^a^	0.001 ± 0.000 ^a^	0.029 ± 0.001 ^c^	0.022 ± 0.001 ^c^	0.015 ± 0.000 ^b^
Dimethyl trisulfide	0.001 ± 0.000 ^a^	0.005 ± 0.001 ^a^	0.014 ± 0.001 ^ab^	0.03 ± 0.003 ^c^	0.036 ± 0.005 ^c^	0.021 ± 0.001 ^b^	0.005 ± 0.000 ^a^	0.004 ± 0.000 ^a^	0.008 ± 0.001 ^a^
1-Octen-3-ol	0.014 ± 0.001 ^a^	0.011 ± 0.000 ^a^	0.025 ± 0.003 ^a^	0.047 ± 0.002 ^b^	0.039 ± 0.006 ^b^	0.05 ± 0.005 ^b^	0.057 ± 0.007 ^b^	0.088 ± 0.006 ^c^	0.113 ± 0.005 ^d^
1-Heptanol	0.011 ± 0.001 ^a^	0.009 ± 0.001 ^a^	0.021 ± 0.001 ^a^	0.045 ± 0.001 ^b^	0.032 ± 0.000 ^a^	0.043 ± 0.001 ^b^	0.052 ± 0.002 ^b^	0.08 ± 0.007 ^c^	0.101 ± 0.014 ^d^
2-Pentyl-Thiophene	0.000 ± 0.000 ^a^	0.000 ± 0.000 ^a^	0.000 ± 0.000 ^a^	0.000 ± 0.000 ^a^	0.000 ± 0.000 ^a^	0.000 ± 0.000 ^a^	0.019 ± 0.002 ^b^	0.000 ± 0.000 ^a^	0.000 ± 0.000 ^a^
2-Furancarboxaldehyde	0.018 ± 0.002 ^f^	0.139 ± 0.017 ^e^	18.762 ± 1.781 ^b^	1.148 ± 0.001 ^d^	36.749 ± 2.82 ^a^	0.406 ± 0.054 ^e^	0.000 ± 0.000 ^c^	1.138 ± 0.027 ^d^	0.000 ± 0.000 ^c^
Benzaldehyde	0.052 ± 0.006 ^d^	0.119 ± 0.01 ^c^	0.158 ± 0.007 ^b^	0.264 ± 0.023 ^a^	0.194 ± 0.009 ^a^	0.203 ± 0.004 ^a^	0.15 ± 0.007 ^b^	0.201 ± 0.01 ^a^	0.267 ± 0.002 ^a^
Benzonitrile	0.000 ± 0.000 ^a^	0.000 ± 0.000 ^a^	0.000 ± 0.000 ^a^	0.000 ± 0.000 ^a^	0.000 ± 0.000 ^a^	0.009 ± 0.001 ^b^	0.000 ± 0.000 ^a^	0.000 ± 0.000 ^a^	0.000 ± 0.000 ^a^
2-Methyl-Propanoic acid	0.004 ± 0.000 ^a^	0.007 ± 0.001 ^b^	0.001 ± 0.000 ^a^	0.01 ± 0.000 ^b^	0.009 ± 0.001 ^b^	0.008 ± 0.001 ^b^	0.008 ± 0.001 ^b^	0.012 ± 0.001 ^b^	0.017 ± 0.002 ^b^
1-Octanol	0.004 ± 0.001 ^a^	0.004 ± 0.001 ^a^	0.009 ± 0.000 ^d^	0.019 ± 0.001 ^c^	0.019 ± 0.002 ^c^	0.021 ± 0.000 ^c^	0.024 ± 0.002 ^c^	0.036 ± 0.000 ^b^	0.043 ± 0.003 ^a^
1-Nonanol	0.000 ± 0.000 ^a^	0.004 ± 0.000 ^b^	0.001 ± 0.000 ^ab^	0.003 ± 0.000 ^b^	0.000 ± 0.000 ^a^	0.003 ± 0.000 ^b^	0.003 ± 0.000 ^b^	0.005 ± 0.000 ^b^	0.008 ± 0.001 ^b^
2-Furanmethanol	0.012 ± 0.001 ^a^	0.018 ± 0.000 ^a^	0.03 ± 0.003 ^b^	0.141 ± 0.015 ^c^	0.000 ± 0.000 ^d^	0.036 ± 0.005 ^b^	0.029 ± 0.004 ^b^	0.01 ± 0.000 ^a^	0.018 ± 0.001 ^a^
Benzeneacetaldehyde	0.187 ± 0.026 ^d^	1.097 ± 0.079 ^a^	1.433 ± 0.127 ^b^	1.519 ± 0.167 ^b^	1.128 ± 0.137 ^a^	0.959 ± 0.081 ^a^	0.271 ± 0.000 ^c^	0.189 ± 0.008 ^d^	0.278 ± 0.032 ^c^
2-Acetylthiazole	0.272 ± 0.008 ^b^	0.115 ± 0.014 ^a^	0.101 ± 0.009 ^a^	0.577 ± 0.05 ^c^	0.192 ± 0.027 ^a^	0.175 ± 0.01 ^a^	0.282 ± 0.006 ^b^	0.089 ± 0.004 ^a^	0.106 ± 0.006 ^a^
2-Thiophenecarboxaldehyde	0.12 ± 0.013 ^a^	0.16 ± 0.014 ^a^	0.415 ± 0.046 ^ab^	0.369 ± 0.033 ^b^	1.271 ± 0.051 ^c^	0.186 ± 0.012 ^a^	0.148 ± 0.008 ^a^	0.189 ± 0.026 ^a^	0.114 ± 0.001 ^a^
Nitro-Benzene	0.000 ± 0.000 ^a^	0.000 ± 0.000 ^a^	0.028 ± 0.002 ^b^	0.000 ± 0.000 ^a^	0.000 ± 0.000 ^a^	0.000 ± 0.000 ^a^	0.000 ± 0.000 ^a^	0.000 ± 0.000 ^a^	0.000 ± 0.000 ^a^
5-Methyl-2-thiophenecarboxaldehyde	0.105 ± 0.01 ^c^	0.205 ± 0.012 ^b^	0.081 ± 0.002 ^c^	0.189 ± 0.009 ^b^	0.093 ± 0.001 ^c^	0.639 ± 0.061 ^a^	0.264 ± 0.027 ^b^	0.196 ± 0.024 ^b^	0.711 ± 0.047 ^a^
Benzeneethanol	0.134 ± 0.014 ^c^	0.189 ± 0.003 ^b^	0.33 ± 0.019 ^b^	0.284 ± 0.028 ^b^	0.295 ± 0.01 ^b^	0.287 ± 0.041 ^b^	0.266 ± 0.007 ^b^	0.402 ± 0.011 ^a^	0.554 ± 0.014 ^a^

Data are presented as mean ± standard deviation. N = 3. Values bearing different lowercase letters in the same row were significantly different (*p* ≤ 0.05) according to Tukey’s post-hoc test.

## Data Availability

The original contributions presented in the study are included in the article. Further inquiries can be directed to the corresponding author.
